# Effects of previous cyclic loading history on subsequent cyclic hehaviour of sand

**DOI:** 10.1371/journal.pone.0331063

**Published:** 2025-08-28

**Authors:** Xiangwu Luo, Wen Song

**Affiliations:** Gansu Province Transportation Planning Survey & Design Institute Co., Ltd, Lanzhou City, China; IGDTUW: Indira Gandhi Delhi Technical University for Women, INDIA

## Abstract

Sand widely employed as subgrade materials, experience varying traffic-induced cyclic loads during service. This study investigates how prior loading history alters their subsequent cyclic deformation characteristics through multistage drained cyclic triaxial tests. Specimens underwent two-phase loading with controlled stress amplitude variations (increase, decrease, or maintenance) in the second stage. Key findings reveal: 1) Stress history critically governs deformation patterns—increased second-stage amplitude reactivates strain accumulation, while hysteresis loop reopening depends on the cumulative plastic strain from first-stage loading; Hysteresis loop reopening in subsequent loading is governed by whether prior deformation exceeds the first-cycle strain threshold observed in virgin sand under equivalent stress amplitude; A novel deformation model incorporating strain accumulation rates and equivalent cycle numbers quantitatively predicts stress-history effects. The proposed framework advances the mechanistic understanding of sand’s memory-dependent cyclic behavior, providing practical tools for infrastructure design under multistage traffic loading conditions.

## 1. Introduction

With the rapid advancement of large-scale infrastructure development in China, particularly in the construction of subways, high-speed railways, airports, and highways, the significance of foundation design and maintenance has become increasingly prominent [1–5]. Sand, as a globally abundant discrete granular material, plays a crucial role in foundation backfilling and land reclamation projects. During its service life, sand-based foundations are often subjected to various cyclic loads, including traffic loading, machinery vibrations, seismic activities, and wave forces. Under such repetitive loading conditions, cumulative strains may develop within the sand, leading to alterations in its dynamic mechanical behavior [6–12]. Consequently, investigating the post-cyclic behavior of sand is essential for evaluating the safety and functionality of foundation engineering projects.

A critical aspect of cyclic soil behavior is the “memory effect,” where soils retain residual effects of prior loading histories. For granular materials, Lopez [13] demonstrated that sand subjected to multi-stage cyclic loading retains a memory of prior stress amplitudes if volumetric strain equilibrium is not achieved, leading to continuity in strain accumulation when re-exposed to similar stresses. Liu [14] further revealed that such equilibrium corresponds to specific stress states and can be disrupted by changes in stress paths, such as horizontal stress reduction during excavation simulations, which accelerates subsequent strain accumulation. Similar memory phenomena extend to cohesive soils: undrained cyclic triaxial tests on kaolin by Liu [15,16] showed that stress disturbances altering deviatoric stress disrupt cyclic equilibrium, triggering sustained pore pressure and strain accumulation. These studies collectively emphasize that cyclic memory—whether in sand or clay—is governed by the interplay between prior stress amplitudes, equilibrium states, and subsequent stress perturbations.

Stress disturbances and reconsolidation processes can destabilize the cyclic equilibrium of soils. For instance, Xia [17] observed that reconsolidation densifies liquefiable sand but also enhances liquefaction resistance by disrupting prior cyclic equilibrium. Similarly, Liu [18] demonstrated that intermittent drainage during multi-stage cyclic loading of kaolin led to greater cumulative deformation than continuous loading, as reconsolidation redistributes particle contacts and forces re-establishment of equilibrium. Analogous findings were reported for fine-grained soils: Nie [19,20] and Li [21] identified that recompaction or stress-path changes in subgrade soils reset cyclic memory, necessitating new equilibrium states and amplifying post-disturbance strain. These results highlight that while reconsolidation improves density, it also erases prior cyclic memory, leading to complex trade-offs in deformation behavior.

In practical scenarios, traffic-induced cyclic stresses exhibit significant amplitude variability. Field measurements by Yang [22] and Li [21] revealed dynamic stress amplitudes ranging from 14 kPa (subway foundations) to 185 kPa (heavy axle loads), while Ma [23] emphasized variations caused by differing subway vehicle types and passenger loads. To address this variability, Li [21] and Tang [24] proposed strain accumulation models based on multi-stage cyclic triaxial tests with ascending stress amplitudes, introducing the concept of “equivalent cyclic times” to account for stress history. However, these models neglect critical aspects: (1) the effects of decreasing cyclic stress amplitudes and (2) explicit mechanisms linking amplitude changes to strain accumulation rates. This gap limits their applicability to real-world conditions where stress amplitudes fluctuate unpredictably. In this study, a series of drained multi-stage cyclic loading tests were conducted on Fujian sand to investigate its deformation behavior under varying cyclic stress amplitudes. Three distinct loading modes were employed: (1) increasing the cyclic stress amplitude in the second loading stage, (2) maintaining a constant cyclic stress amplitude across stages, and (3) decreasing the cyclic stress amplitude. The effects of altering the cyclic stress amplitude on the deformation behavior of sand were systematically analyzed and discussed. These tests provide valuable insights into how changes in cyclic stress amplitude influence the mechanical response and cumulative deformation of sand under multi-stage cyclic loading conditions.

## 2. Sample preparation and test program

### 2.1. Test apparatus and sample preparation

An advanced dynamic triaxial test system (DYNTTS) was used to perform cyclic triaxial tests in the study. The system was manufactured GDS Instruments Ltd. UK. More descriptions of the system can be seen in Liang [25].

**Fig 1:**
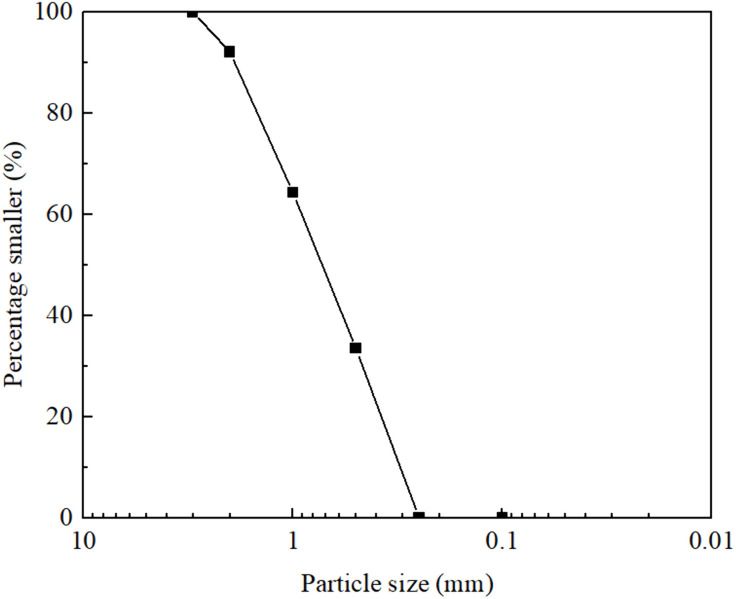
Grain-size distribution curve of Fujian sand.

The test material used in this study was Fujian sand with a specific gravity of 2.64. The maximum and minimum void ratios of the sand are 0.71 and 0.32, respectively. The selected sand exhibits a mean particle size (d_50_) of 0.78 mm and an effective internal friction angle of 33.9°. The particle size distribution curve can be seen in Fig 1.

To eliminate the influence of sample preparation methods on test results, all specimens in this study were prepared using the dry deposition method. The oven-dried sand was divided into five equal portions based on the target relative density. Each portion was slowly poured into the mold through a funnel in layers, with each layer controlled to a height of approximately 20 mm. A rubber hammer was used to gently tap the mold’s sidewalls to compact the sand and achieve the desired specimen height. Prior to mold removal, a vacuum pressure of approximately −20 kPa was applied using a vacuum pump to verify airtightness and stabilize the specimen. After demolding, the actual dimensions of the specimen were measured (final dimensions: diameter = 50 mm, height ≈ 100 mm, yielding a height-to-diameter ratio of h/d ≈ 2) and used as the baseline for subsequent testing.

A combined approach of CO₂ replacement, water head saturation, and back-pressure saturation was employed to fully saturate the triaxial specimens. After filling the confining chamber with water, the confining pressure was increased to 30 kPa, and the vacuum pressure was released by opening the top and bottom valves. CO₂ gas was then injected from the specimen’s bottom to displace internal air, with the gas exiting through the top. This CO₂ replacement process lasted approximately 15 minutes to ensure complete air displacement. Subsequently, de-aired water was introduced from the bottom of the specimen until a steady, bubble-free outflow was observed at the top, confirming the completion of water head saturation. To further enhance saturation, back-pressure saturation was conducted after water head saturation. Both back pressure and confining pressure were incrementally increased while maintaining an effective confining pressure of 20 kPa. In this study, a back pressure of 400 kPa was applied to all specimens. All specimens exhibited a Skempton’s B-value >0.96, meeting the saturation requirements for the experiments.2.2 Test program

Following saturation, specimens underwent a two-stage consolidation process: (1) isotropic consolidation under 50 kPa effective confining pressure, followed by (2) anisotropic consolidation through deviatoric stress application until reaching a stress ratio *K* = 0.5. All tests were conducted on specimens with comparable relative densities (*D*_*r*_ ≈ 0.75), where *D*_*r*_ is defined as:


$$D_{r}=\frac{e_{\max}-e}{e_{\max}-e_{\min}}$$ (1)

in which *e*_*max*_, *e*_*min*_ are the maximum and minimum void ratios of Fujian sand, respectively.

Following consolidation, specimens underwent the first-stage cyclic loading comprising 500 one-way sinusoidal cycles at 0.05 Hz under drained conditions. The selected frequency aligns with prior studies [24–28] demonstrating negligible frequency effects (0.05–30 Hz range) on sand deformation. This low-frequency protocol ensures complete excess pore water pressure dissipation while maintaining mechanical response comparability to higher-frequency loading scenarios. The cyclic stress amplitudes range of 30–70 kPa were adopted, which is smaller than 185 kPa (axle load of 25 tons) reported by Li [21] but similar to 20–80 kPa used by Liang [25].

**Fig 2:**
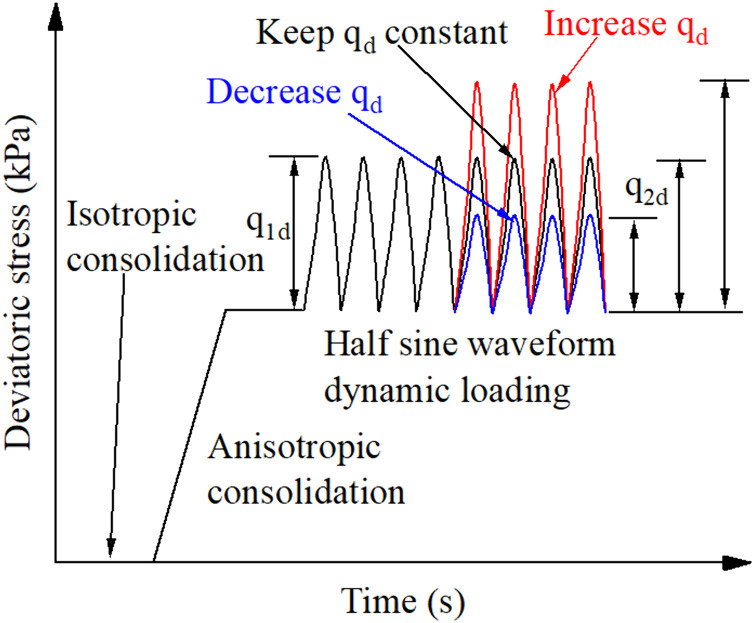
Schematic diagram of cyclic loading.

After completing the first-stage cyclic loading, all specimens were subjected to an additional 500 cycles of one-way sinusoidal loading at the same frequency of 0.05 Hz. The second-stage loading amplitude was intentionally varied relative to the first stage (as illustrated in Fig 2), creating three distinct scenarios: increased amplitude, decreased amplitude, or unchanged amplitude compared to the initial loading phase. This controlled variation enabled systematic investigation of how prior stress history influences subsequent deformation patterns under amplitude transitions.

Table 1 summarizes the experimental program of cyclic triaxial tests. Specimens were systematically labeled using the notation CCq_1d_-q_2d_, where q_1d_ and q_2d_ denote the cyclic deviator stress amplitudes (kPa) applied during the first and second loading stages, respectively. For instance, the specimen CC30–50 underwent sequential cyclic loading with amplitudes of 30 kPa (Stage I) and 50 kPa (Stage II). This nomenclature explicitly encodes stress history parameters, enabling direct correlation between specimen labels and their multistage loading protocols.

**Table 1:** The experimental program of cyclic triaxial tests.

Test series	Test ID	*D*_*r*_ (%)	σ_3_ (kPa)	N_1_ (times)	N_2_ (times)	q_1d_ (kPa)	q_2d_ (kPa)
1	CC30−30	≈75	50	500	500	30	30
	CC30–50						50
	CC30–70						70
2	CC40-40					40	40
	CC40-60						60
3	CC50−50					50	50
	CC50–60						60
	CC50–70						70
4	CC60−40					60	40
	CC60−50						50
	CC60–70						70
5	CC70−50					70	50
	CC70−60						60
	CC70−70						70

## 3. Results and analysis

This section presents the effects cyclic loading history on axial strain accumulation, volume strain accumulation and resilience modulus of sand.

### 3.1. the cyclic behaviour of sand without cyclic loading history

**Fig 3:**
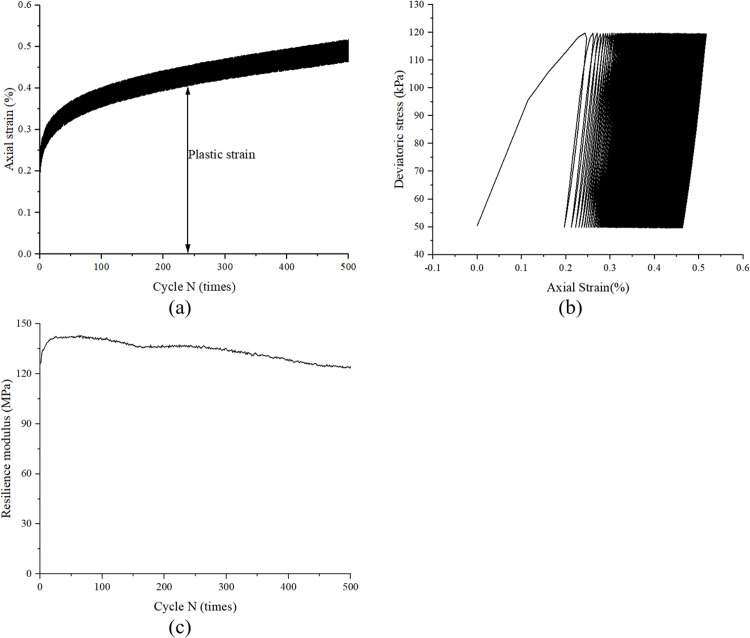
Typical behaviour of sand under drained cyclic loading, obtained from sample CC70−70.

Fig 3 illustrates the typical cyclic response of sand under drained conditions, as observed in sample CC70−70. As depicted in Fig 3a, significant axial strain accumulation occurs during the initial stage of cyclic loading, followed by a gradual stabilization with reduced accumulation rate in subsequent cycles. For instance, the plastic strain exhibited a substantial increase from 0% to 0.31% during the initial 50 loading cycles, whereas only a marginal increment of 0.03% was observed between cycles 450 and 500. As the number of cycles increased, the hysteresis loops demonstrated a distinct transition from an unclosed to a closed configuration, as illustrated in Fig 3b. Notably, the slope of the unloading path remained relatively constant throughout the cyclic loading process. This phenomenon can be more clearly observed in Fig 3c, which demonstrates that the resilience modulus initially increased during the early stage of cyclic loading, primarily attributed to the densification effect. Subsequently, the modulus stabilized and maintained a nearly constant value with minor fluctuations throughout the remaining loading cycles. This observed behavior is consistent with the findings reported by Liu [12].

**Fig 4:**
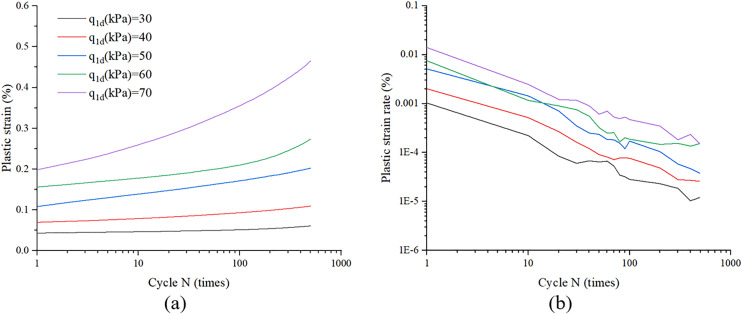
Plastic strain and plastic strain rate versus cycle N under different cyclic stress amplitude.

Plastic strain, defined as the irrecoverable deformation during cyclic loading, is illustrated in Fig 3a. Fig 4a and 4b present the characteristic patterns of plastic strain accumulation and its corresponding rate as a function of cycle N under varying cyclic stress amplitudes. As illustrated in Fig 4a, a direct correlation exists between cyclic stress amplitude and plastic strain accumulation during initial loading cycles. Specifically, higher stress amplitudes induce greater plastic strain magnitudes accompanied by accelerated strain accumulation rates. Furthermore, Fig 4b demonstrates a progressive attenuation in plastic strain rate with increasing cycle count. Notably, when analyzed in logarithmic coordinates, specimens subjected to varying stress amplitudes exhibit comparable decay rates in plastic strain rate development.

This study establishes a quantitative framework to predict plastic strain accumulation in sand under varying cyclic stress conditions. The proposed model combines initial cyclic loading strain with progressive plastic strain accumulation rates, as formalized in Equation (2).


$$\varepsilon_{a1}^{p}(N_{1})=\varepsilon_{a1}^{p}(0)+\sum_{1}^{N_{1}}{\dot{\varepsilon }}_{a1}^{p}(N)$$ (2)

Where $\varepsilon_{a1}^{p}(N_{1})$ is the plastic deformation of the specimen after *N*_*1*_ cyclic loading. Subscript 1 indicates the first stage cyclic loading; $\varepsilon_{a1}^{p}(0)$ is initial cyclic loading plastic strain; ${\dot{\varepsilon }}_{a1}^{p}(N)$ is the plastic strain rate of *N*th to (*N* + 1)th cyclic loading.

As claimed by Cai [29], the initial cyclic loading plastic strain can be a function of cyclic stress amplitude, as shown in equation (3).


$$\varepsilon_{a1}^{p}(0)=\alpha_{1}{\left(\frac{q_{1d}}{p_{atm}}\right)}^{\beta_{1}}$$ (3)

**Fig 5:**
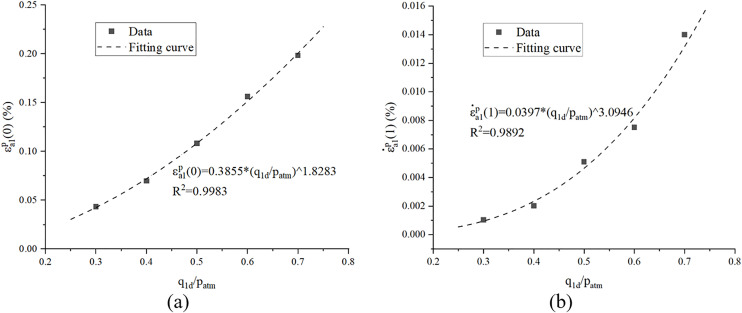
Model parameter determination.

Where α_1_and β_1_ are constants related to soil properties; $p_{atm}$ is standard atmospheric pressure. Through regression analysis, we can see that in this paper that α_1_ = 0.3855, β_1_ = 1.8283, as illustrated in Fig 5a.

As illustrated in Fig 4b, the plastic strain rate exhibits a decaying trend with increasing cycle count, while the slopes of the curves for specimens subjected to distinct cyclic stress amplitudes demonstrate comparable magnitudes within the log-log coordinate regime. Thus, equation (4) can be adopted to predicted the plastic strain rate.


$$\begin{array}{c} \ln{\dot{\varepsilon }}_{a1}^{p}(N)=\ln{\dot{\varepsilon }}_{a1}^{p}(1)+k\ln N\\ {\dot{\varepsilon }}_{a1}^{p}(N)={\dot{\varepsilon }}_{a1}^{p}(1)*N^{k} \end{array}$$ (4)

Where ${\dot{\varepsilon }}_{a1}^{p}(1)$ is plastic strain rate of cycle 1–2; *k* is a constant representing the attenuation of plastic strain rate. In the study, *k* = −0.7013 was adopted.

It must be pointed out that the ${\dot{\varepsilon }}_{a1}^{p}(1)$ rather than ${\dot{\varepsilon }}_{a1}^{p}(0)$ or $\varepsilon_{a1}^{p}(0)$ were used as initial value to calculate plastic strain rate due to excessive strain magnitudes generated during the first loading cycle, as evidenced in Fig 4a, rendering these data points unsuitable for analytical purposes of plastic strain rate.


$${\dot{\varepsilon }}_{a1}^{p}(1)=\alpha_{2}{\left(\frac{q_{1d}}{p_{atm}}\right)}^{\beta_{2}}$$ (5)

Where α_2_and β_2_ are constants related to soil properties and α_2_ = 0.0397, β_2_ = 3.0946 were adopted through regression analysis, as illustrated in Fig 4b.

The methodological foundation of excluding initial plastic strain in strain rate calculations stems from two critical considerations: (1) The initial strain inherently encapsulates complex interactions from specimen preparation variability and stress history effects, which disproportionately dominate the strain rate calculation when retained (2) Stage II initial strain demonstrates threshold-dependent behavior may modulated by Stage I accumulation, necessitating their decoupling to isolate the true cyclic deformation mechanism.

**Fig 6:**
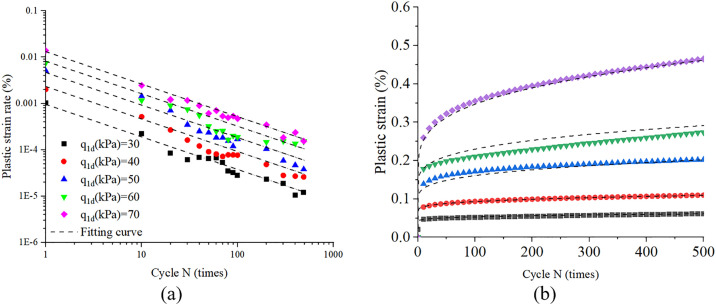
Plastic strain and plastic strain rate versus cycle N, comparison of tested data and model predicted value.

Fig 6a and 6b illustrate the evolution of plastic strain and plastic strain rate with cycle N under different cyclic stress amplitude conditions as predicted by Equation (2), with experimental data superimposed for comparison. As shown in these figures, the model demonstrates favorable predictive performance, effectively capturing strain accumulation in sand under drained cyclic loading conditions.

### 3.2. the cyclic behaviour of sand with cyclic loading history

**Fig 7:**
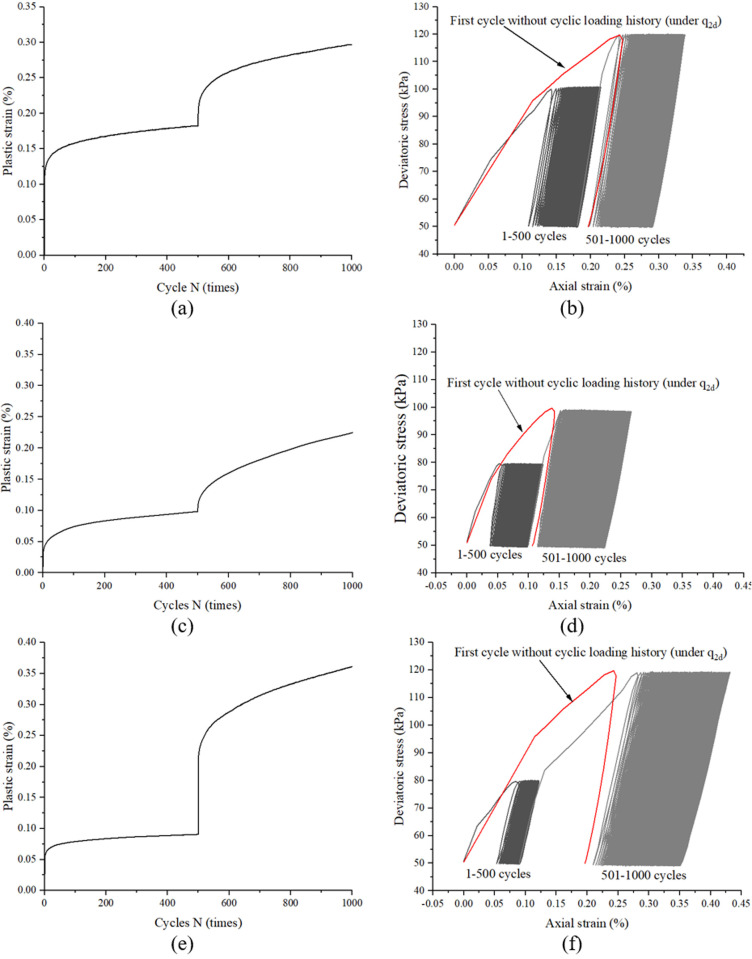
Typical response of under a two-stage cyclic loading protocol with significantly incrementally increased stress amplitudes, obtained from sample CC50-70 (a and b), CC30-50 (c and d) and CC30-70 (e and f).

Fig 7a and 7b depict the deformation response under a two-stage cyclic loading protocol with incrementally increased stress amplitudes, using experimental data from specimen CC50–70 (stage I: 50 kPa; stage II: 70 kPa). As observed from Fig 7a and 7b, the specimen exhibits a decelerating strain accumulation pattern after 500 cycles in stage I (50 kPa cyclic loading). However, upon transitioning to stage II with a 40% higher cyclic stress amplitude (70 kPa), renewed strain accumulation occurs accompanied by the reopening of stress-strain hysteresis loops, as explicitly captured in Fig 6b. The progressively decreasing strain accumulation rate indicates that the sand specimen attains a stable internal configuration under 50 kPa cyclic loading; whereas renewed rapid strain development during stage II (70 kPa cyclic loading) provides mechanistic evidence of retained adjustment capacity within the soil fabric. Crucially, such structural reorganization manifests only under notably elevated stress amplitude excitation. Two additional representative specimens (CC30–50 and CC30–70) are presented in Fig 7c–7e, f respectively, demonstrating behavioral characteristics similar to specimen CC50–70. Notably, the stress-strain curves of the first cycle without prior cyclic stress history (under q_2d_ conditions) are systematically compared in Fig 7b, 7d, 7f. Despite specimen preparation variations, all cases consistently exhibit: 1. The total accumulated strain in Stage I remains smaller than the initial cycle strain in Stage II under non-cyclically preloaded conditions; 2. Renewed strain accumulation emerges during the first cycle of Stage II.

**Fig 8:**
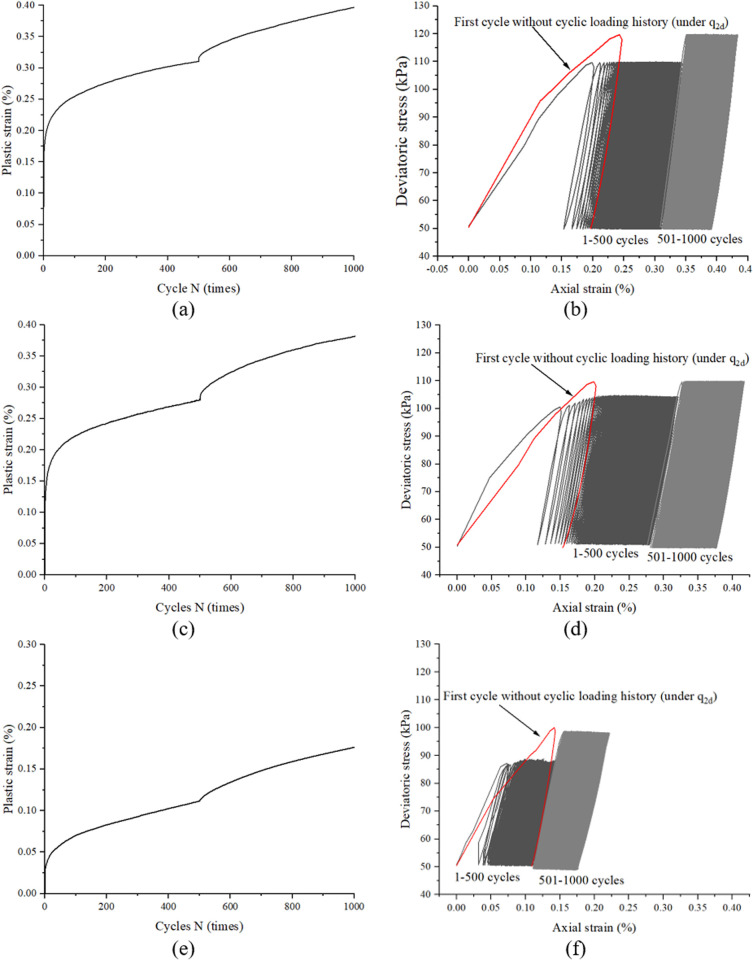
Typical response of under a two-stage cyclic loading protocol with slightly incrementally increased stress amplitudes, obtained from sample CC60-70 (a and b), CC50-60 (c and d) and CC40-50 (e and f).

Fig 8a and 8b present the strain response of specimen CC60–70 subjected to a two-stage cyclic loading protocol (stage I: 60 kPa; stage II: 70 kPa). In contrast to specimen CC50–70, three critical distinctions emerge: (1) the strain accumulation rate during stage II in CC60–70 decreases by 38% compared to CC50–70 (quantified via derivative analysis), (2) the stress-strain hysteresis loops remain essentially closed during initial stage II cycles, and (3) the cumulative strain increment in stage II constitutes only 22% of that observed in CC50–70. These phenomena suggest that the 60 kPa pre-conditioning induces a metastable structural configuration, wherein the subsequent 16.7% stress amplitude increase (60 → 70 kPa) becomes insufficient to overcome the enhanced cyclic resistance threshold established during stage I loading. Two additional representative specimens (CC50–60 and CC40–50) are presented in Fig 8c–8e, f respectively, demonstrating analogous behavioral patterns to specimen CC60–70. For systematic comparison, Fig 8b, 8d, f explicitly include the stress-strain curves of the first cycle without prior cyclic stress history under q_2d_ conditions. Despite inherent variability induced by specimen preparation, the following consistent trends are observed: 1. The total accumulated strain in Stage I systematically exceeds the initial cycle strain of Stage II under non-precycled conditions; 2. Negligible strain accumulation occurs during the first cycle of Stage II.

**Fig 9:**
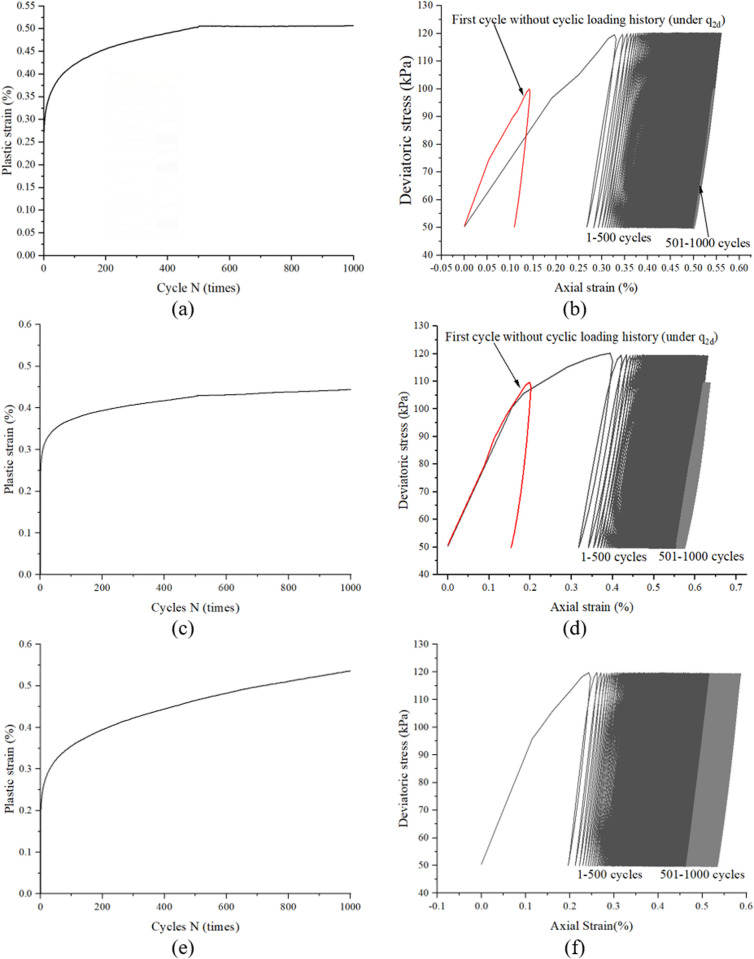
Typical response of under a two-stage cyclic loading protocol with detrimentally increased stress amplitudes, obtained from sample CC70−50 (a and b), CC70−60 (c and d) and keep stress amplitudes constant, obtained from sample CC70−70.

Fig 9a and 9b display the experimental results from specimen CC70−50 under a two-stage cyclic loading sequence with reduced amplitude in stage II (stage I: 70 kPa; stage II: 50 kPa). Notably distinct from the response observed in Fig 7, three key features emerge: (1) complete closure of stress-strain hysteresis loops persists throughout stage II, (2) strain accumulation ceases entirely loading stage II (plastic strain increment < 0.01%), and (3) the unloading-reloading curves exhibit near-perfect superposition. This mechanical behavior strongly indicates that the 70 kPa pre-consolidation in stage I has established a cyclically stable structure, rendering the subsequent 28.6% stress amplitude reduction (70 → 50 kPa) incapable of reactivating plastic deformation mechanisms. Two additional representative specimens (CC70−60 and CC70−70) are systematically presented in Fig 9c–9f, demonstrating a critical phenomenon under this specific loading configuration: measurable strain accumulation is nearly absent throughout Stage II.

The phased loading experimental results presented in Figs 7−9 demonstrate three characteristic responses governed by prior cyclic stress history:(1) Significant stress amplitude increase: Reopening of primary hysteresis loops with subsequent accelerated strain accumulation (e.g., CC50−70, CC30−50 and CC50−70 in Fig 7); (2) Moderate stress amplitude increase: Minimal hysteresis loop development accompanied by decelerating strain accumulation (e.g., CC60−70, CC50−60 and CC40−50 in Fig 8); (3) Stress amplitude reduction and kept constant: Complete hysteresis loop closure and strain accumulation cessation (e.g., CC70−50, CC70−60 and CC70−70 in Fig 9). These observations align with yet extend existing theoretical frameworks: While López’s cyclic memorability theory [13] explains the stabilized response under drained conditions, and Liu’s stress perturbation concept [14] describes stability breakdown mechanisms, our findings specifically reveal that critical stress amplitude escalation suffices to reactivate plastic deformation processes. This threshold behavior provides experimental validation for the existence of a metastable “cyclic hardening envelope” in sand, where post-stabilization deformation reactivation depends nonlinearly on subsequent stress amplitude increments.

To quantitatively predict the cyclic deformation response of sand with prior loading history, a refined classification of the three characteristic responses is essential. As demonstrated in Fig 7b, specimen CC50–70 subjected to 500 cycles at 50 kPa in stage I accumulates a total strain of 0.18%, which remains below the first-cycle strain (0.2%) of virgin sand under 70 kPa (refer to Fig 5a). This sub-threshold preconditioning results in hysteresis loop reopening upon stage II loading initiation. Conversely, specimen CC60–70 develops a stage I cumulative strain of 0.31% (exceeding the 0.2% first-cycle threshold of virgin sand at 70 kPa), which effectively suppresses hysteresis loop development in stage II (Fig 8b). This critical observation establishes a strain threshold criterion for hysteresis loop activation: when $\varepsilon_{a1}^{p}(N_{1})<\varepsilon_{a2}^{p}(0)$ hysteresis reactivation and when $\varepsilon_{a1}^{p}(N_{1})\geq \varepsilon_{a2}^{p}(0)$, hysteresis suppression. This criterion mechanistically links prior deformation history to subsequent cyclic responses, providing a quantitative basis for predicting hysteresis behavior in multi-stage loading scenarios.

The experimental observations can be mechanistically explained through the interplay between cyclic stress history and granular structural evolution. During stage I cyclic loading with lower stress amplitudes (e.g., 50 kPa), sand undergoes progressive particle rearrangement and fabric optimization. These microstructural adjustments govern subsequent responses under higher stresses, as plastic deformation inherently reflects irreversible fabric reorganization. Crucially, repeated low-amplitude cycles can induce structural modifications comparable to those caused by fewer high-amplitude cycles. When the preconditioning-induced structural adaptation is incomplete (as in specimen CC50–70), the first cycle of stage II loading primarily completes this pending adjustment process. The stress-strain curve initially follows the memory path of stage I unloading until exceeding the prior stress threshold (50 kPa), beyond which renewed particle sliding drives further strain accumulation (Fig 7b). Conversely, when preconditioning achieves sufficient structural adaptation (as in CC60–70), the fabric has already stabilized to accommodate the subsequent loading magnitude. In this scenario, the stage II loading curve maintains mechanical consistency with the optimized structure, bypassing the adjustment phase and proceeding directly to subsequent deformation accumulation (Fig 8b). This threshold-dependent behavior reveals a fundamental mechanism: The first cycle of stage II loading serves either as a structural completion phase (if preconditioning is inadequate) or a direct deformation phase (if preconditioning is sufficient). The transition between these two modes is governed by the relative magnitude of accumulated plastic strain during preconditioning versus the inherent deformation demand of the subsequent stress level.

**Fig 10:**
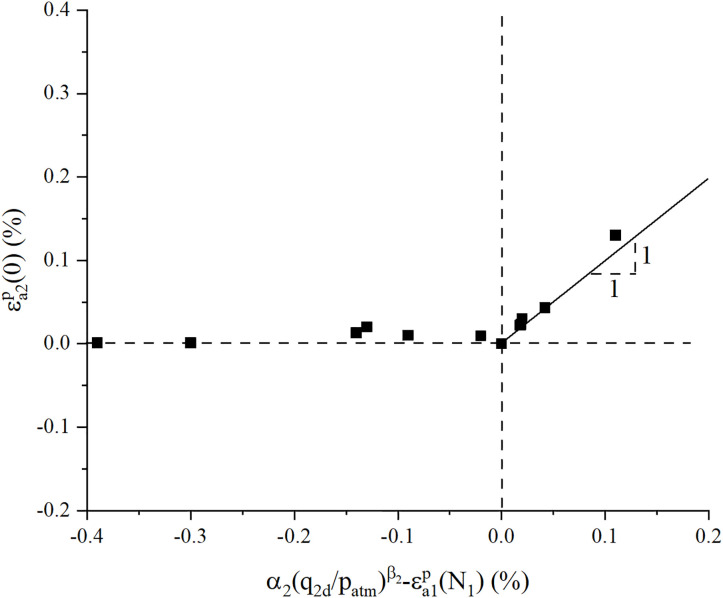
Initial cyclic strain in Stage II.

Fig 10 demonstrates the critical relationship between $\alpha_{2}{\left(\frac{q_{2d}}{p_{atm}}\right)}^{\beta_{2}}-\varepsilon_{a1}^{p}(N_{1})$ -values and the initial cyclic strain in Stage II, revealing two distinct mechanical regimes: negligible strain accumulation when $\alpha_{2}{\left(\frac{q_{2d}}{p_{atm}}\right)}^{\beta_{2}}-\varepsilon_{a1}^{p}(N_{1})$ < 0 versus a linearly proportional increase with $\alpha_{2}{\left(\frac{q_{2d}}{p_{atm}}\right)}^{\beta_{2}}-\varepsilon_{a1}^{p}(N_{1})$ -values under positive $\alpha_{2}{\left(\frac{q_{2d}}{p_{atm}}\right)}^{\beta_{2}}-\varepsilon_{a1}^{p}(N_{1})$ conditions. This threshold-dependent behavior conclusively validating the deformation mechanism framework proposed in our analysis.

Based on the above theoretical framework, the initial plastic strain of Stage II $\varepsilon_{a2}^{p}(0)$ can be expressed by:


$$\varepsilon_{a2}^{p}(0)=\max\left\{0,\;\alpha_{2}{\left(\frac{q_{2d}}{p_{atm}}\right)}^{\beta_{2}}-\varepsilon_{a1}^{p}(N_{1})\right\}$$ (6)

Equation (7) effectively formulates the initial cyclic strain induced by stress history effects, where parameter $\varepsilon_{a2}^{p}(0)$ exhibits threshold-dependent behavior: $\varepsilon_{a2}^{p}(0)$ = 0 when $\alpha_{2}{\left(\frac{q_{2d}}{p_{atm}}\right)}^{\beta_{2}}-\varepsilon_{a1}^{p}(N_{1})$ < 0, transitioning to $\varepsilon_{a2}^{p}(0)$ = $\alpha_{2}{\left(\frac{q_{2d}}{p_{atm}}\right)}^{\beta_{2}}-\varepsilon_{a1}^{p}(N_{1})$ when $\alpha_{2}{\left(\frac{q_{2d}}{p_{atm}}\right)}^{\beta_{2}}-\varepsilon_{a1}^{p}(N_{1})$ > 0. This piecewise formulation physically represents the critical stress ratio ($\alpha_{2}{\left(\frac{q_{2d}}{p_{atm}}\right)}^{\beta_{2}}-\varepsilon_{a1}^{p}(N_{1})$ =0) as the threshold governing the activation of initial plastic strain under cyclic loading histories.

**Fig 11:**
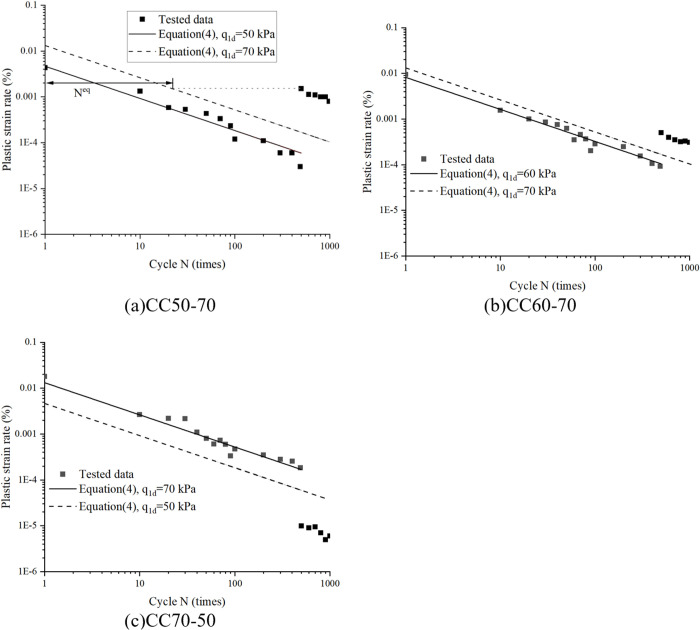
Plastic strain rate versus cyclic N under different loading conditions, comparison of tested data and model predicted value. (c)CC70−50.

Fig 11 illustrates the relationship between plastic strain rate and cycle number for the tested specimens, with theoretical predictions from Equation 4 superimposed. As can be seen in Fig 11a, after 500 cycles in stage I, the decay rate of plastic strain rate under modified stress amplitudes aligns closely with that of virgin sand under equivalent loading conditions, as evidenced by the parallel slopes of experimental and predicted curves. However, experimental curves systematically lag behind theoretical predictions, manifesting as a rightward displacement. This hysteresis effect quantifies the “cyclic memory” embedded in sand’s fabric through prior loading history. To operationalize this observation, we introduce the concept of equivalent cycle number (*N*^*eq*^), defined as history-compensated cycle (after equivalent cycles, the two-stage strain accumulation rate reaches equal to that after loading stage 1), as illustrated in Fig 11. It is determined by equating the accumulated plastic strain from stage I to the strain that would develop under stage II loading over *N*^*eq*^ (both excluding the initial cycle). This approach transforms the prediction challenge into a cycle-number mapping problem, where historical loading effects are translated into equivalent deformation progress through strain-energy equivalence principles, bypassing complex constitutive modeling while preserving physical interpretability.

Through above analysis, *N*^*eq*^ can be determined by equation (8):


$$\begin{array}{c} \sum_{1}^{N^{eq}}{\dot{\varepsilon }}_{a2}^{p}(N)=\sum_{1}^{N_{1}}{\dot{\varepsilon }}_{a1}^{p}(N)\\ {\dot{\varepsilon }}_{a2}^{p}(N)={\dot{\varepsilon }}_{a2}^{p}(1)*N^{k}\\ {\dot{\varepsilon }}_{a2}^{p}(1)=\alpha_{2}{\left(\frac{q_{2d}}{p_{atm}}\right)}^{\beta_{2}} \end{array}$$ (7)

Simplification is available:


$$\sum_{N=1}^{N=N^{eq}}N^{k}={\left(\frac{q_{1d}}{q_{2d}}\right)}^{\beta_{2}}\sum_{N=1}^{N=N_{1}}N^{k}$$ (8)

**Fig 12:**
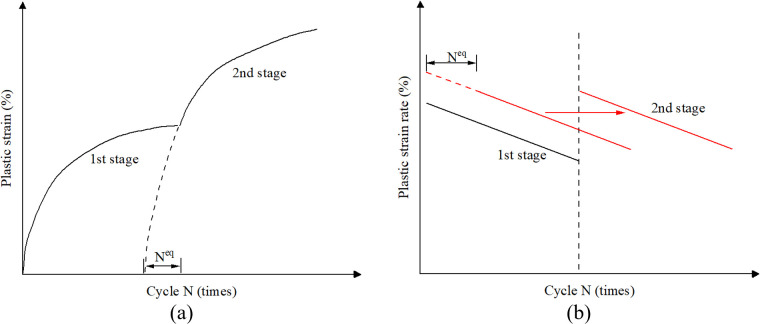
Equivalent cycle number and its action effect.

Regarding the Stage II initial plastic strain rate, while the analysis identifies two distinct cases about initial strain, the predictive model does not require their separate consideration. As demonstrated in Fig 11, the Stage II accumulated plastic strain rate exhibits the same characteristic decay rate as Stage I (evidenced by parallel slopes in the logarithmic plot), irrespective of the specific pattern. The critical difference lies solely in the initial value at the onset of Stage II. This initial value is conceptualized as the plastic strain rate predicted by the third term of Equation (8) after undergoing *N*^*eq*^ equivalent cycles under the Stage II stress level, rather than for a virgin state. Consequently, the model’s focus shifts to determining *N*^*eq*^. This is achieved via the strain-equivalence method expressed in the first term of Equation (8), which intrinsically incorporates the magnitude of prior strain accumulation from Stage I. Therefore, the influence of the prior loading history, manifesting as the two distinct initial rate cases, is comprehensively accounted for within the single framework of the *N*^*eq*^ parameter. This parameterization inherently captures the effect of accumulated pre-strain on the Stage II initial condition, rendering separate classification within the model unnecessary (Fig 12).

The plastic strain of loading stage 2 can be expressed by:


$$\varepsilon_{a2}^{p}(N_{2})=\varepsilon_{a2}^{p}(0)+\int_{N^{eq}+1}^{N^{eq}+N_{2}}{\dot{\varepsilon }}_{a2}^{p}(N)dN$$ (9)

Crucially, the accumulated plastic strain during Stage II was calculated by integrating the plastic strain rate from cycle *N*^*eq*^ + 1 to *N*^*eq*^ + *N*, rather than from cycle 1 to *N*. This approach inherently accounts for the influence of prior cyclic stress history on the Stage II plastic strain rate.

The total plastic strain of loading stage 1 and 2 can be expressed by:


$$\varepsilon_{a}^{p}(N_{1}+N_{2})=\varepsilon_{a1}^{p}(0)+\int_{1}^{N_{1}}{\dot{\varepsilon }}_{a1}^{p}(N)dN+\varepsilon_{a2}^{p}(0)+\int_{N^{eq}+1}^{N^{eq}+N_{2}}{\dot{\varepsilon }}_{a2}^{p}(N)dN$$ (10)

**Fig 13:**
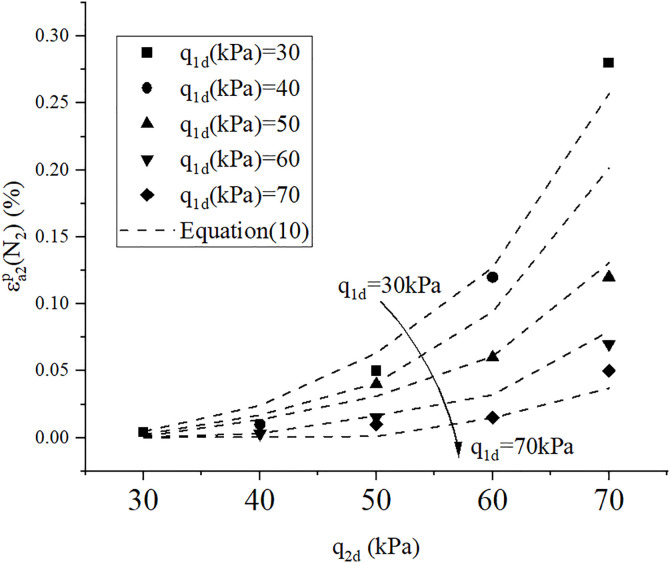
Experimental results with model predictions usingEquation (10).

Fig 13 compares experimental results with model predictions for multi-stage cyclic loading under various conditions. The close alignment between measured data and theoretical curves demonstrates the model’s strong predictive capability in characterizing the deformation behavior of sand under drained multi-stage cyclic loading. The model accurately captures the strain accumulation retardation effect induced by prior cyclic hardening, particularly evident in specimens with high-stage preconditioning. This validation confirms that the proposed framework effectively integrates stress history effects, amplitude-dependent hardening, and cycle-induced fabric evolution, providing a robust tool for analyzing complex multi-stage cyclic loading scenarios in geotechnical engineering practice.

## 4. Conclusions

To investigate the stress-history effects on saturated sand under drained cyclic loading, this study conducted multistage cyclic triaxial tests with controlled amplitude variations (increase, decrease, or maintenance) in secondary loading phases. Key conclusions emerge:

1. The tested Fujian sand demonstrates favorable subgrade characteristics, exhibiting limited cumulative deformation (<1%) under high-cycle loading at 70 kPa stress amplitude with 75% relative density, suggesting its engineering suitability for transportation embankments.
2. Initial plastic strain rate shows exponential dependence on cyclic stress amplitude. Plastic strain rate decay follows linear patterns in log-log coordinates, with decay rates (slopes) remaining stress-amplitude-independent.
3. Stage-I loading triggers significant hardening through particle rearrangement and force-chain restructuring, reducing Stage-II deformation and Hysteresis loop activation in Stage-II, which is controlled by a threshold mechanism: prior accumulated strain must exceed the first-cycle strain threshold of virgin sand under equivalent stress. This reveals how stress history modulates deformation by altering stable fabric states.
4. Although stress history reduces Stage-II strain accumulation rates, the preserved logarithmic decay rates identical to virgin sand indicate that cyclic preconditioning modifies initial fabric conditions (e.g., force-chain distribution, void ratio) rather than fundamentally altering the inherent particle-scale deformation physics (sliding-rolling mechanisms).
5. A deformation model incorporating equivalent cycle numbers successfully predicts post-history cyclic responses.

The conclusions are derived from Fujian sand testing. Generalizability to other sands requires validation through comparative mineralogical studies. Subsequent research should explore undrained conditions and complex loading sequences.
